# Nanoparticle Near-Surface Electric Field

**DOI:** 10.1186/s11671-016-1258-3

**Published:** 2016-02-01

**Authors:** Levan Chkhartishvili

**Affiliations:** Department of Engineering Physics, Georgian Technical University, Merab Kostava Avenue 77, 0175 Tbilisi, Georgia; Laboratory for Boron and Powdered Composite Materials, Ferdinand Tavadze Institute of Metallurgy and Materials Science, Alexander Kazbegi Avenue 15, 0160 Tbilisi, Georgia

**Keywords:** Surface reconstruction, Near-surface electric field, Nanomaterial

## Abstract

Theoretical studies show that surface reconstruction in some crystals involves splitting the surface atomic layer into two—upper and lower—sublayers consisting of atoms with only positive or only negative effective electric charges, respectively. In a macroscopic crystal with an almost infinite surface, the electric field induced by such a surface-dipole is practically totally concentrated between the sublayers. However, when the material is powdered and its particles are of sufficiently small sizes, an electric field of a significant magnitude can be induced outside the sublayers as well. We have calculated the distribution of the electric field and its potential induced at the surface of a disc-shaped particle. The suggested novel nanoscale effect explains the increase in physical reactivity of nanopowders with decreasing particle sizes.

## Background

Examining spontaneous surface reconstruction which leads to the formation of a dipole layer at the free crystalline surface, let us consider the surface layer of hexagonal boron nitride (h-BN) which is a layered crystal. In h-BN, intralayer bonding is strong being mainly covalent and partially ionic—B and N atoms have positive and negative effective charges, respectively—while interlayer bonding being of van der Waals type is weak. Accordingly, in h-BN, the length of intralayer bonds is more than two times shorter than that of interlayer bonds. Therefore, atoms can be rather easily displaced in the direction perpendicular to the layers than within the layers.

Thus, in h-BN, the most probable surface reconstruction is the displacement of different atoms in opposite directions from the surface hexagonal layer. Indeed, in [1], the study of the structural properties of single-layer h-BN by molecular dynamics (MD) demonstrated that the lowest energy surface defect in single-layer h-BN involves out-of-plane displacement of a nitrogen atom to form a tetrahedron with three boron atoms in the plane. Evidently, both types of displacements of all the nitrogen atoms and all the boron atoms in the opposite directions are equivalent to the reconstruction of the surface atomic layer into two parallel sublayers consisting of atoms with opposite atomic charges.

The same is true for boron nitride nanotubes, which are hexagonal layers wrapped into cylinders. Using the MD method, it was shown in [2] that relaxation of their structure results in a wave-like or “rippled” surface, in which B atoms rotate inward and N atoms outward, reminiscent of the surface relaxation of the bulk III–V compounds.

The electrostatic potentials on both the outer and the inner surfaces of single-walled BN nanotubular model systems were computed in [3] within the Hartree–Fock (HF) approach. The BN tubes were found to have strong and variable surface potentials, and their inner surfaces were markedly positive.

Thus, one can conclude that surface reconstruction in boron nitrides with layered (both flat and tubular) structures involves splitting the surface atomic layer into two, upper and lower, sublayers, which consist of atoms with negative and positive effective charges, respectively. It implies the formation of an ultra-thin dipole layer at the free surface. The same should be true for any other layered binary crystal, as well as for any material consisting of atoms with non-zero static effective atomic charges.

Recently, applying extensive and highly accurate ab initio simulations for electron dynamics under laser illumination by solving the time-dependent Schrödinger equation of electrons and simultaneously Newton’s equation of ions-motion, it was found that the exposure of h-BN to an IR laser of a certain wavelength triggers vibrations in the lattice with B and N atoms moving in opposite directions [4]. Since these atoms have effective electric charges of opposite signs, such vibrations originate the dynamic dipoles. The possibility of formation of a dynamic dipole layer from a single hexagonal layer in h-BN crystal serves for one more argument for the possibility of static dipole layer formation from the free-surface layer in result of its reconstruction.

In the case of an infinite crystalline surface, the macroscopic electric field induced by such a surface-dipole is almost totally concentrated between sublayers, but when the material is powdered into particles of sufficiently small sizes, one should also expect the emergence of a near-surface electric field of a significant strength. The same effect is expected for nano-sized step-like islands on crystalline surface and nanoporous materials.

Here, we calculate the approximated distributions of the electric field and its potential induced at the surface of the particle with a dipole surface layer. The suggested novel nanoscale effect explains the frequently observed increase in physical reactivity of nanopowders with the increase in their dispersity.

## Methods

Usually, opposite charges of crystal surface sublayers are related to opposite static charges of constituting atoms. Particularly, in h-BN, a positive charge is associated with boron (B) atom and a negative one with nitrogen (N). As is known, it is very hard to measure atomic charges. Almost all physical properties of the material via its electronic structure are related to the atomic charges; however, these relations are so complicated that it is impossible to determine by them the values of the effective charges. That is why we have developed [5, 6] a method of the semiempirical estimation of atomic charges in binary compounds on the basis of empirical parameters (number of atomic pairs in the unit cell, lattice constants, Young’s modulus, and dielectric constant). Note that in general, effective charges are crystallographic-direction-dependent. In particular, in h-BN, intra- and interlayer effective atomic charge numbers are found to be 0.35 and 0.09, respectively.

Another necessary step in the estimation of the surface-dipole layer effect is to make a realistic assumption about the shape of particles of the material in its powdered form. In particular, for h-BN nanopowders, we have already developed [7–9] a morphology model and proposed a disc-like shape with a fixed aspect ratio *η* = *r*/*h*, where *h* is the thickness of the disc and *r* is its radius.

For h-BN, the aspect ratio of nanoparticles was estimated empirically as *η* = 5.32. This value is very close to the ratio of squares of inter- and intralayer B–N bonds lengths, *D* ≈ 3.3306 A and *d* ≈ 1.4457 A, respectively: *D*^2^ / *d*^2^ ≈ 5.31, and practically coincides with the theoretical value of $9\;\left(3+2\sqrt{2}\right)\;/\;{\pi}^2\approx 5.32$, which follows from the geometric model for boron nitride-layered nanosystems [10]. This coincidence seems not to be accidental as, according to the well-known Harrison’s interpolation scheme [11], any energy-parameter of a crystal has to be inversely proportional to the square of the corresponding length-parameter. Thus, the aspect ratio for h-BN nanoparticles equals to the ratio of inter- and intralayer binding energies per B–N bond.

The additional argument is provided by a number of experimental images of h-BN nanoparticles (see e.g., [12–14]) distinctly demonstrating their disc-like shapes.

## Results and discussion

In order to calculate the near-surface electric field based on the surface-dipole layer and particle morphological models described above, we consider space distribution of the electric field induced by two parallel atomic layers with opposite charges.

**Fig. 1:**
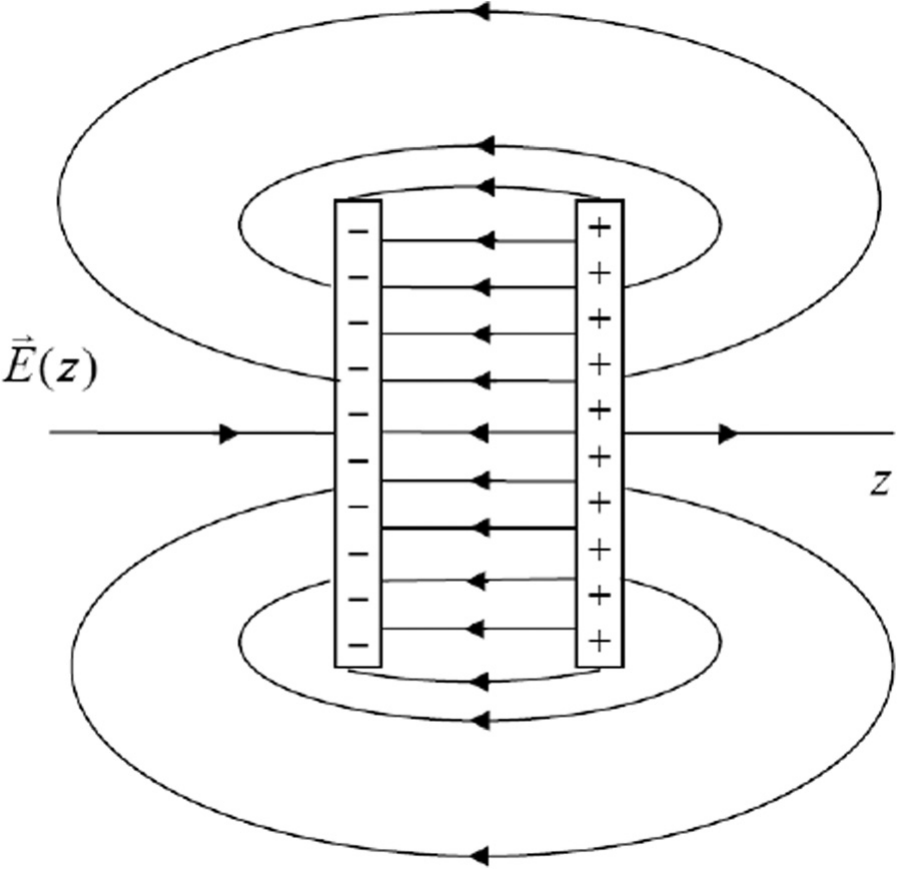
Schematic view of electric field line distribution near the dipole layer

From the schematic view (Fig. 1), we see that in the plane parallel to the surface the electric field lines density is approximately uniform. So, we can restrict ourselves by calculating the dependence of the electric field potential *φ* on the distance *z* from the surface, i.e., in the *z*-direction (perpendicular to the surface plane) if we consider that the upper sublayer is charged positively.

**Fig. 2:**
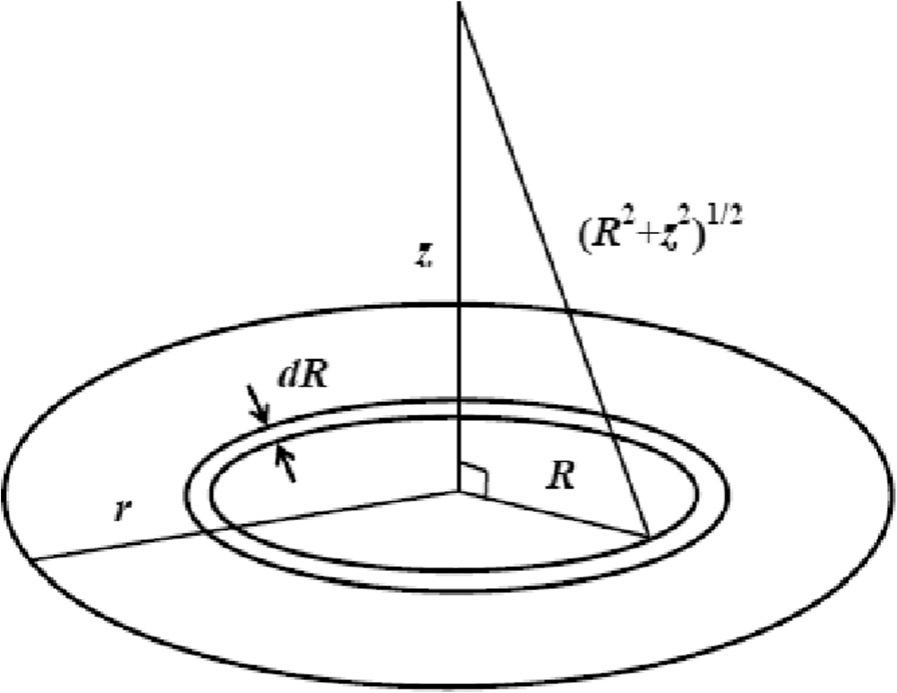
Elementary flat dipole on the surface of a disc-like particle

Since the particle is assumed to be disc-shaped, it is convenient to choose elementary surface-dipole (Fig. 2) in the form of a flat ring of radius *R* and elementary width dR with 0 ≤ *R* ≤ *r*, where *r* is the disc radius. The ring-like area of such elementary dipole is

1 $$ \mathrm{d}\mathrm{S}=2\pi\;R\mathrm{d}\mathrm{R}. $$

If the surface density of dipole moment is *σ*, then the elementary dipole moment equals to

2 $$ \mathrm{d}\mathrm{p}=2\pi \sigma\;R\mathrm{d}\mathrm{R}. $$

It is convenient to calculate *φ*(*z*) at a distance *z* from the disc center, where the potential of the electrical field produced by the elementary dipole is

3 $$ \mathrm{d}\varphi =\frac{\varphi z\;R\mathrm{d}\mathrm{R}}{2{\varepsilon}_0\;{\left({z}^2+{R}^2\right)}^{3/2}}\;. $$

Then, the electric potential on the *z*-axis produced by the whole disc-shaped dipole is found to be

4 $$ \varphi (z)=\frac{\sigma z}{2{\varepsilon}_0}\;{\displaystyle \underset{0}{\overset{r}{\int }}\frac{R\mathrm{d}\mathrm{R}}{{\left({z}^2+{R}^2\right)}^{3/2}}.} $$

Finally, we derive explicitly the near-surface distribution of the electric field potential *φ* = *φ*(*z*) induced by the surface-dipole layer of disc-shaped particle:

5 $$ \frac{\varphi (z)}{\varphi_0}\approx 1-\frac{1}{{\left(1+\frac{r^2}{z^2}\right)}^{1/2}}, $$

where

6 $$ {\varphi}_0\equiv \varphi (0)=\frac{\sigma }{2{\varepsilon}_0} $$

is the height (depth) of the near-surface potential barrier (well) for positively (negatively) charged species.

From the relation *E* = − dφ / dz, we can find the near-surface electric field strength as

7 $$ \frac{E(z)}{E_0}\approx \frac{1}{{\left(1+\frac{z^2}{r^2}\right)}^{3/2}}, $$

where

8 $$ {E}_0\equiv E(0)=\frac{\sigma }{2{\varepsilon}_0r} $$

denotes the maximum of the near-surface field strength.

**Fig. 3:**
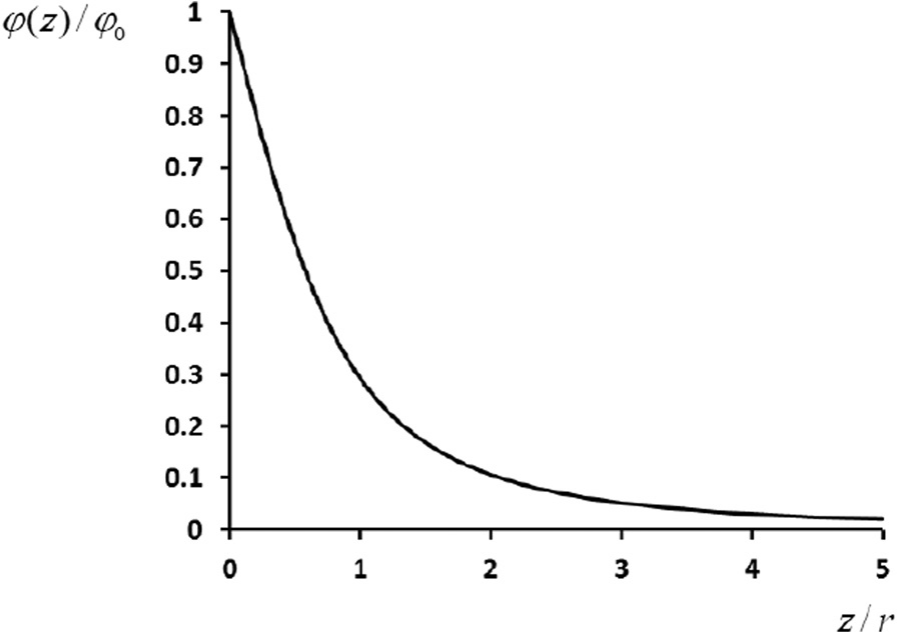
Electric field potential versus distance from the nanoparticle surface

**Fig. 4:**
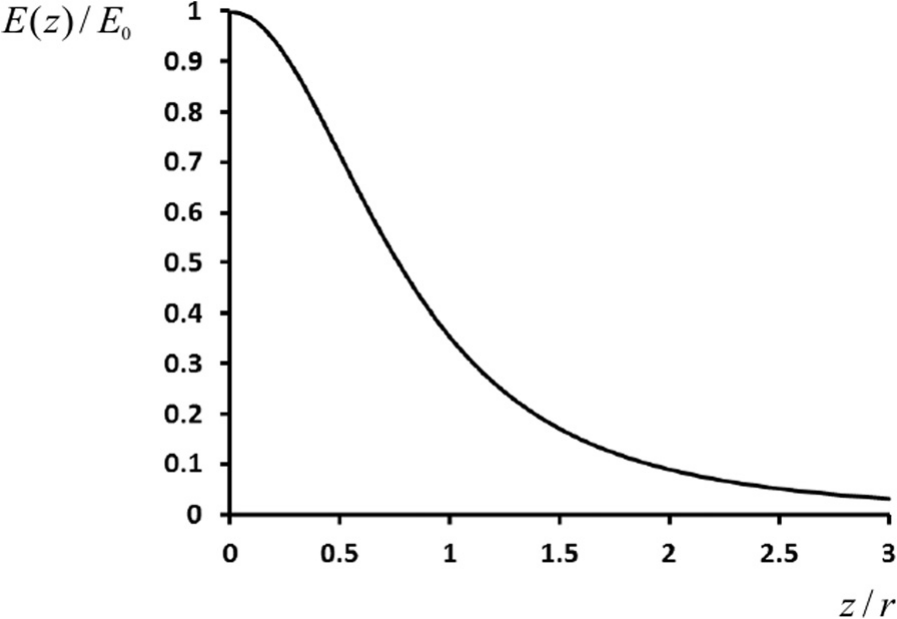
Electric field strength versus distance from the nanoparticle surface

The functions *φ* = *φ*(*z*) and *E* = *E*(*z*), obtained for the near-surface electric field, are presented in Figs. 3 and 4, respectively.

Our model implies that we consider the electric field near-surface layer at distances greater than the distance between the atoms which form the two oppositely charged sublayers of the reconstructed surface. Usually, this condition is satisfied as the obtained near-surface electric field potential induced by the surface-dipole layer should be added by the Pauli repulsive potential in the form of a well of almost infinite height placed at a certain distance *z*_0_ which exceeds interatomic distances of both the intra- and inter-sublayers as, in general, the Pauli repulsion radius *z*_0_ is a sum of half-thickness of the dipole layer, the van der Waals atomic radius of the upper sublayer, and the radius of a particle (an atom, molecule, cluster, and corresponding ion) interacting with the surface.

The suggested mechanism of inducing the near-surface electric field is a true nanoscale effect because at the limit of infinite surface,

9 $$ r\to \infty \kern0.48em , $$

the potential function tends to a constant, zero in our case, value

10 $$ \varphi (z)\to 0\kern0.36em , $$

i.e., electric field strength vanishes as well,

11 $$ E(z)\to 0\kern0.48em . $$

## Conclusions

Summarizing the above analysis, we can state that the weakness of the interaction between atomic layers parallel to a crystal free-surface makes it possible reconstruction of the surface layer when different atoms are displaced in opposite directions from the surface plane. If bonding is partially ionic, i.e., different atoms possess non-zero effective electrical charges of opposite sign, under the reconstruction, the surface atomic layer polarizes and creates a non-zero electric field near the surface of finite particles of the crystalline material. Thus, the suggested novel nanoscale effect explains the enhancement in physical reactivity of nanopowdered/nanoporous materials with decreasing of particles/porous sizes (e.g., see recent report [15] where porous boron nitride microfibers are found to be an effective material capturing pollutants from aqueous solutions).
